# Green Walls Could Cut Street-Canyon Air Pollution

**DOI:** 10.1289/ehp.121-a14

**Published:** 2013-01-01

**Authors:** Rebecca Kessler

**Affiliations:** Rebecca Kessler, based in Providence, RI, writes about science and the environment. She is a member of the National Association of Science Writers and the Society of Environmental Journalists.

Rows of tall buildings can create a unique urban habitat known as a street canyon. These canyons trap traffic pollutants, limiting their dispersal into the atmospheric boundary layer that extends as high as 2,000 meters above the ground. A new study suggests that vegetation in street canyons may reduce air-pollutant concentrations much more than previously reported and suggests innovative planting configurations to improve city pollution hot spots.^1^

Outdoor air pollution is believed to cause an estimated 1.3 million annual deaths worldwide,^2^ as well as an increased risk of respiratory and cardiovascular diseases.^3^ Plantings are often promoted as a partial solution, because leaves absorb gaseous pollutants through their pores and capture particulate matter on their surfaces.^4^^,^^5^^,^^6^^,^^7^^,^^8^^,^^9^^,^^10^^,^^11^ Yet modeling studies of the vegetation across entire cities have estimated that existing green cover reduces air pollution concentrations by less than 1.5%.^4^^,^^5^^,^^9^

For the current study, researchers developed a computer model to calculate how much pollution is captured by vegetation in the much smaller, somewhat isolated space of a street canyon. “We argue that for urban air quality these effects will be much more important because people aren’t found five hundred meters up in the atmosphere; they’re found down at street level,” says lead author Thomas Pugh, a postdoctoral researcher in atmospheric chemistry now at the Karlsruhe Institute of Technology.^12^

The team estimated that street-canyon vegetation may reduce concentrations of two of the most harmful urban air pollutants, nitrogen dioxide (NO_2_) and coarse particulate matter (PM_10_), by as much as 40% and 60% respectively, although average reductions over a year were in the range of 7–30%. Because air lingers in street canyons, it stays in contact with pollutant-scrubbing vegetation, Pugh says.

David Nowak, who studies how urban forests affect environmental quality for the U.S. Forest Service, likens the effect to that of an air purifier running in a small, enclosed room as opposed to a large, open space. He points out that the new results are not entirely unexpected—at least one other study showed similar reductions in the air pollutant ozone, although those measurements were made in an urban forest, not a city.^5^

The study is limited by the model’s reliance on data with only modest experimental support, including the rates at which plants capture pollutants and air flows in and out of street canyons, says Pugh. Moreover, experimental research in vegetated street canyons is needed to verify the results. This lack of validation makes Max Zhang, an associate professor of engineering at Cornell University who studies traffic emissions, question the size of the pollutant reductions the paper reports. “I still believe the argument is very good,” says Zhang, “I believe there are definitely reductions, but the problem is the magnitude.”

Nevertheless, Pugh says city planners may be able to design plantings that significantly improve air quality in highly polluted street canyons. The model results suggest that plants growing vertically on building walls could remove nearly 10 times as much NO_2_ and nearly 12 times as much PM_10_ from street-canyon air as horizontally grown rooftop vegetation. The researchers even propose adding “green billboards” to rooftops to increase the time polluted air spends within a canyon, maximizing its exposure to vegetation.

**Figure:**
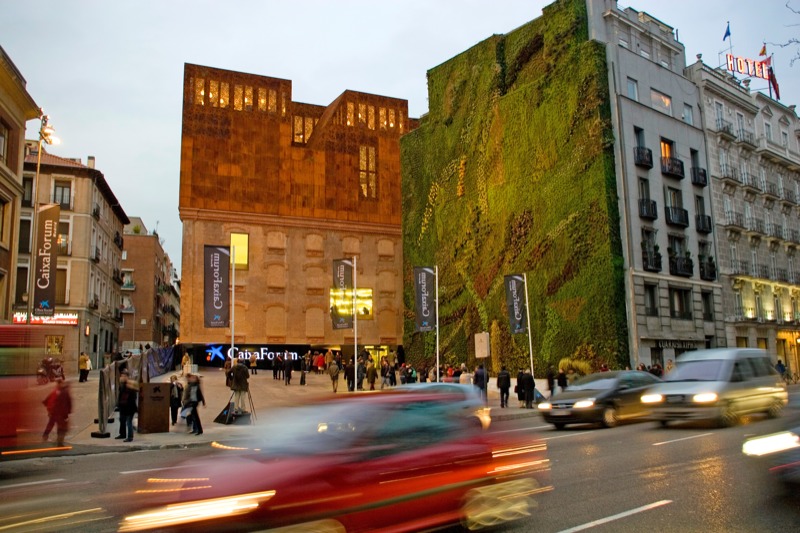
Vertical plantings may be a better option than trees or green roofs for mitigating street-canyon pollution. © Ismael Alonso/Cover/Getty Images

Whether green billboards or green walls are practical on a large scale remains to be seen. Walter Warriner, community forester for the city of Santa Monica, California, and a board member of the National Urban and Community Forestry Advisory Committee, is unaware of any plantings specifically targeted at urban pollution hot spots in the United States. But he says they may soon be possible given advances in air-quality monitoring technology and a recent focus among urban foresters on quantifying environmental benefits.

Trees are a more familiar solution, but although Pugh and colleagues did not directly model how trees capture airborne pollutants,^13^ they predict that in highly polluted street canyons, trees may actually do more harm than good. That’s because in those circumstances, trees’ ability to capture pollutants may be outweighed by their tendency to trap vehicle emissions near street level, right where people can breathe them in.^14^^,^^15^

“That’s not to say you should go and chop down all the trees in busy street canyons,” says Pugh, but planners contemplating planting new trees in these settings should proceed with caution to make sure they don’t inadvertently increase ground-level pollution while trying to address some other issue, such as rainwater runoff or beautification. Where traffic is light, trees offer clear benefits, the researchers write.

Nowak notes that although plants can certainly help reduce urban air pollution, reducing emissions is a more effective, if not necessarily an easy, first step. In the worst cases, he says, “We’re not going to plant our way out of this problem.”
